# Change in Psychotherapy: A Dialogical Analysis Single-Case Study of a Patient with Bulimia Nervosa

**DOI:** 10.3389/fpsyg.2012.00546

**Published:** 2012-12-07

**Authors:** Alessandro Salvini, Elena Faccio, Giuseppe Mininni, Diego Romaioli, Sabrina Cipolletta, Gianluca Castelnuovo

**Affiliations:** ^1^Department of Philosophy, Sociology, Education and Applied Psychology, University of PaduaPadova, Italy; ^2^Department of Education, Psychology and Communication, University of BariBari, Italy; ^3^Department of Philosophy, Pedagogy and Psychology, University of VeronaVerona, Italy; ^4^Department of General Psychology, University of PaduaPadova, Italy; ^5^Psychology Research Laboratory, Istituto Auxologico Italiano IRCCS, Ospedale San GiuseppeVerbania, Italy; ^6^Department of Psychology, Catholic University of MilanMilan, Italy

**Keywords:** dialogical self, I-positions, motivation for change, psychotherapy, discourse analysis, relational perspective

## Abstract

Starting from the critical review of various motivational frameworks of change that have been applied to the study of eating disorders, the present paper provides an alternative conceptualization of the change in psychotherapy presenting a single-case study. We analyzed six psychotherapeutic conversations with a bulimic patient and found out narratives “for” and “against” change. We read them in terms of tension between dominance and exchange in I-positions, as described by Hermans. These results indicate that the dialogical analysis of clinical discourse may be a useful method to investigate change from the beginning to the end of therapy.

## Introduction

Existential difficulties and psychological disorders bring states of malaise which sometimes become so serious as to arouse the wish to modify the conditions causing them, through psychotherapeutic intervention. Indeed, the theoretical proposition capable of summing up the plausibility of any psychotherapeutic treatment is “You need to change.” However, this need for change does not guarantee satisfaction because it depends on the variable and sometimes conflicting meanings of change.

In the literature, various motivational approaches have been developed in an attempt to prevent patients from dropping out of treatment, to increase their active engagement and, hence, to improve the short-term and long-term outcome of therapy. We shall discuss the theme of change departing from the analysis of different conceptualizations of motivation for change and introducing some semiotic positions.

## Approaches to Motivation

### The will is to say: “I can start again”

Arendt (1978) analyzed the will as a springboard for action and as an “organ of the future.” She illustrated how this faculty to trigger something new, and so to “change the world,” can function in the world of appearances. The comparison among various philosophical positions – from Epictetus to Duns Scotus, from Stuart Mill to Nietzsche (1882) – led this student of Heidegger (1982) to attempt to overcome Kant’s rather awkward definition of the will as the power to spontaneously originate a series of successive things or states. Arendt (1978) noted that just as thinking prepares the self for a role as a spectator, so the will shapes it into a “durable self” which orients all the single acts of the will. Since the will creates the character of the self, we are able to interpret it as principium individuationis: the source of the person’s specific identity. However, she continued, this very individualization as produced by the will creates a serious new problem for the idea of “freedom.” Shaped by the will and aware that he might be different from what he/she is (unlike body appearance, talents, and aptitudes, the character is not produced by the self at birth), the individual always tends to assert a “myself” opposed to an indeterminate “they”: all the others who I as an individual am not. Arendt (1978) believed that nothing can be more frightening than a solipsistic notion of freedom (p. 523).

The recommendation to rely on “men of action” more than “professional thinkers” (philosophers or scientists) clearly shows that freedom (political freedom, not philosophical) is an “attribute not of I-want, but of I-can” (Arendt, 1978, p. 528). Political action takes shape in the individuals’ awareness that since they have only limited power available to them, they can aspire only to a limited freedom. The problem involved in “men of action,” i.e., persons who want to “change the world” (if only “their” inner world), is the enigma of the beginning, which puts them “face to face with the abyss of liberty.” To be open to change means to acknowledge that “the very nature of any beginning is to bear an element of complete arbitrariness” (Arendt, 1978, p. 535). Change induced by the beginning of something – whatever that thing might be – implies that it might not have been and, at the same time, once produced, that it can no longer be destroyed (at least not totally). The experience of conversion, marvelously told in Augustine’s *Confessions*, favored the intuition that self-change represents the will to be reborn; this awareness values the fragile liberty of the beginnings, of the initiative.

This overview on the seminal theory of will proposed by Arendt aims at supporting a dialogical view of change. According to this viewpoint, change follows the specific Self’s rhetoric of the “beginnings” which founds human liberty. Resistance to change, instead, derives from the slavery of repeating, which traps the dialogical Self. The tension between “change” (liberty of reborn) and “resistance to change” (self-determination to repetition) can be also represented as voices discussing and contrasting in the context of a personal arena, in the dynamic of a dialog between Parts (“the Selves”). These varying manifestations of the will can be discovered in certain “psycho-discursive practices” which characterize specific interpretative repertoires of subjectivity (Greimas, 1983; Gergen, 2009). Psychotherapy represents also a privileged laboratory to investigate and discover exchange in the relationship between voices, and also to understand in which way people change, amplifying the role of some voices and reducing the impact of others (Wetherell, 2008; Faccio et al., 2012b).

### Telling himself/herself to will something

In the transition from modern to post-modern psychology, change-oriented motivation may be inserted in a dialogical conception of the “self.” From our research standpoint, the expression of a thought or intention, the utterance of a sentence or the doing of a deed, do not arise from previously well-formed, orderly cognitive processes at the center of our being. Instead, they originate amidst a person’s vague, diffuse, unordered feelings: from their sense of how, semiotically, “they are ‘positioned’ in relation to the others around them” (Shotter, 1993a, p. 63). As James (1890) wrote “feelings of tendency” are “signs of direction” in thought. In this sense, feeling is a phenomenon of the semiotic threshold, uniting the person with his or her surroundings.

The concept of “semiotic position” recalls Vygotsky’s (1962) idea that signs mediate all higher mental activities. Researchers need to expand upon the nature of signs that serve to establish, maintain, and alter the position. Words do have the power to position the speaker with regard to his or her addressees. Finding themselves in a newly assigned position, the discourses will then create responses in order to express their “feeling” about the position and, perhaps, to move into a new position: one that they find more appropriate.

In light of a critical review of literature on this theme (Vygotsky, 1962; Bakhtin, 1981, 1984; Volosinov, 1987; Shotter, 1993a,b; Cheyne and Tarulli, 1999), we shall discuss several suggestions regarding the analysis of semiotic positions through discourse analysis. In particular, we shall take into account implications pertinent to the psychological practice (Vidotto et al., 2006; Romaioli et al., 2008).

## What is Change? How Do People Change?

In everyday speech, “change” is seen as a contrast to permanence; the two are considered as complementary opposites. Nevertheless, identity implies both change and permanence. “Change” and “non-change” narratives are strictly linked to motivational rhetoric expressing *will* and *determination*: “he or she is not ready for change” “he or she hasn’t decided to change yet,” “unless he or she decides to change, no one can help him/her.”

This assumption is the basis for many theoretical models that explore the theme of change; we shall briefly mention some of them; in particular we chose four of the most cited models in the literature on change in psychotherapy.

Miller and Rollnick (1991) defined motivation as “the probability that a person will enter into, continue, and adhere to a specific change strategy” (p. 19). The authors developed a motivational interview (MI) which yielded cumulative insights aiming to help clients become proactive participants in therapy. They assumed that clients possess a powerful potential for change, the clinician’s task is to evoke and strengthen this inner resourcefulness, thereby enhancing the intrinsic motivation for change which is inherent in the individual. This inner growth process is facilitated when the clinician skilfully applies the following four key principles: expressing empathy, developing discrepancy, increasing self-efficacy, and rolling with resistance.

Inspired by the MI of Miller and Rollnick (1991); Vitousek et al. (1998) proposed the Socratic method, which is well known in cognitive therapy as a tool for enhancing motivation for change. The approach involves being empathetic toward the patient’s experiences, as reflected in his acknowledgment of the symptoms’ possible functions, and recognizing that changing one’s behavior is a difficult task. The therapist offers an encouraging framework so that patients can reach conclusions on their own concerning the origin of their symptoms, or the pros and cons of change. The basic assumption is that when a decision to change one’s behavior is experienced as being personally taken rather than imposed by the therapist, the effects of the actual behavioral change will be more lasting.

Another popular approach is the trans-theoretical model of change (TMC; Prochaska and DiClemente, 1982; DiClemente, 1999). The primary goal of this model is to describe the different stages through which patients advance in their movement toward lasting change. People are said to move from pre-contemplation (not considering change at all), to contemplation (weighing the pros and cons of change), to preparation (getting ready to make the change), to action (making the change), and to maintenance (consolidating the positive change). This change process is considered to be cyclical rather than linear in nature. Clinicians can help patients to reach higher-level stages by increasing their internal (or intrinsic) motivation as opposed to their external (or extrinsic) motivation toward change.

The Self-Determination Theory (SDT, by Ryan and Deci, 2000) is a theory of human motivation and personality. It focuses primarily on the quality of motivation, claiming that two different types of high-quality motivation can be distinguished: intrinsic motivation and internalized extrinsic motivation. An adequate analysis of motivational dynamics might well take into account the degree to which the change has been internalized rather than being experienced as pleasurable or exciting (i.e., intrinsic motivation). Although an activity might be initiated by the person rather than by external pressures, some types of internal motivation are less likely to yield lasting benefits because the behavioral regulation is insufficiently anchored within people’s value structures. It is also necessary to investigate the degree to which the change represents a true expression of patients’ personal values (identification) rather than being instigated by internal obligations (introjection). SDT also considers the quality of motivation for change, alongside the quantity.

Although they represent different approaches, these clinical models share three fundamental theoretical presuppositions (Romaioli et al., 2008):

1. Motivation is intended as a cognitive quality, whose persistence is ensured by means of predominantly intra-psychic heuristics (the individualistic proposition); and the act of evaluation is regarded as a calculated choice based on important, essentially logical rules. In this concept of motivation, every action must have its psychological antecedents (whether beliefs, desires, or intentions), which have a cause-effect relationship with the behavior itself. As Searle (2001) critically noted, “there is a long tradition in philosophy (and psychology) according to which in the case of rational action, if the psychological antecedents of the act are all in order, that is, they are the right kind of desires, intentions, value, judgment, etc., then the act must necessarily follow” (p. 220). Observers usually interpret a lack or decrease in motivation (as in relapses) simply as “weakness of the will” (Elstrup, 2009). No possibility is provided that people may act paradoxically, performing actions which are not in accordance with their best judgment, the so-called “akrasia” (Vidotto et al., 2006, 2010; Romaioli et al., 2008; Faccio, 2011, 2012; Faccio et al., 2011a). In everyday life we find numerous examples of such “will inconsistency,” often designated as the “akratic phenomenon”; for instance: If someone really wants to quit smoking, why do they persist in lighting up after a meal? If a person is madly in love with someone, how in the world can they be unfaithful, leave them, or hurt them?
2. Carrying out an action against one’s own will is assumed to be the effect of an impulse which makes an individual give in to temptation; in conversation (a few minutes after the fall) the lapse may translate into a personality trait: “laziness,” “starvation,” or “weak will.” No one considers the possibility that in the past, a person might have desired something different from what he/she now desires, since he/she had different thoughts and motivations. Memories from the past are reconstructed by adapting them to the present. Without the context and the situation which generated an event, however, can we really reconstruct its original meaning? Memories are not fixed photographs, they are mobile. It is not enough to have good memory in order to have reliable memories. Even the past changes, for its configuration depends on our present feeling and narratives (Faccio et al., 2012a; Romaioli and Contarello, 2012; Romaioli and Faccio, 2012; Castiglioni et al., 2013).
3. The third issue is a conceptualization of “change” as if it were an object. Often, we are unaware of the conditions which make us change. In most cases, even after change, it is very difficult to reconstruct the process of change. There is not “a sure route to change,” but there are many narratives for representing change which are consistent with the theory that the person believes in. We know little of change as it occurs in our lives, but we can produce wonderful narratives about it. What appears is a linear path, because we believe in the permanence of meanings. In other words, we imagine the mind as being universal and unchangeable. Bruner (1987, 1991), in speaking about the relationship between experience and the narration of experiences, noted that when a person tells a story, he arbitrarily imposes a logical sense and logical meanings on the stream of consciousness, highlighting some events and ignoring others (Lacasa et al., 2005). Therefore, narratives construe the world according to the narrative style we use to describe it. Change derives from the theories about it, and even therapists will stick to the narratives about change that they believe in, just as patients will choose their own narratives.

### “Change” and “non-change” as the effect of a dialog between voices

In line with a narrative perspective, Hermans (1996, 2001); Hermans et al. (1993) proposed a decentralized conception of the self as multi-voiced and dialogical. More specifically, they defined the dialogical self in terms of a “dynamic multiplicity of I-positions, or voices in the landscape of the mind, intertwined as this mind is with the minds of other people. Positions are not only ‘internal’ (e.g., I as a man, white, Catholic) but also ‘external,’ belonging to the extended domain of the self (e.g., my wife, my children, my colleagues)” (Hermans et al., 1993, p. 78). Dialogs may take place between or among internal positions (e.g., a conflict between my position as a father and my position as a hardworking scientist), between internal and external positions (e.g., I discuss our shared project with my colleague John), and between or among external positions (e.g., disagreement among my teachers on religious topics). The dialogical self is not only part of the broader society, but also functions, itself, as a “society of mind” with tensions, conflicts, and contradictions as intrinsic features of a (healthily functioning) self (Hermans, 2002). Building on the views of figures like Bakhtin (1981, 1984); James (1890); Mead (1934), we envision a multi-voiced dialogical self involved in internal interchanges between I-positions that desire change, and I-positions that oppose change (Ecker and Hulley, 2000, 2008; Cipolletta et al., 2010; Cipolletta, 2011, in press).

### What is dominance between voices?

In common sense the notion of dialog differs from the notion of dominance. Usually, dialog evokes an image of people discussing their views and problems as perfectly equal partners (Hermans et al., 1993). For any dominance to arise in such a situation, it is merely the power of arguments that counts. Such a conception of dialog, however, truly applies only to an ideal dialogical situation. In apparent opposition to this image, Linell (1990) has argued that asymmetry (or dominance) exists in each single act–response sequence: the actors continually alternate the roles of “power holder” and “object of power” in the course of their dialog. As long as one party speaks, the other party is required to be silent. As long as the dominant party talks, the subordinate party allows his or her contributions to be directed, controlled, or inhibited by the interlocutor’s moves (interactional dominance).

Moreover, one party can predominantly introduce and maintain topics and perspectives on topics (topic dominance). The amount of talk reflects dominance relationships as well: the party who talks a lot prevents the other party from taking his turn. Finally, the speaker who makes the most strategic moves may have a strong impact on a conversation without needing to talk a lot. In other words, although the topic of a meaningful conversation is under mutual control, relative dominance is not extrinsic but rather intrinsic to the dialogical process.

The more symmetrical the dialogs is, the more opportunity it provides for mutual influence; the more asymmetrical it is, the more it constrains the exchange of views and experiences. From a clinical point of view the excessive dominance of one voice over another may be a dysfunctional characteristic of the dialogical self (Dimaggio, 2006). A voice may become dominant for a long period of life. As Hermans et al. (1993) remind us in citing Linell (1990), the dominance in interaction is multidimensional. There are many ways in which a party can be said to “dominate,” that is, to control the “territory” shared by the interactants in communication.

### “I and the other part of me who doesn’t want me to change”: A clinical example

As an example, we shall now consider a clinical case. Carlotta is a 23 years old woman diagnosed with an anorexic disorder of bulimic subtype. She’s studying at the university, with the desire to become an archeologist and is living with her parents and two brothers. She started starving herself and lost 8 kg during the previous 2 years. At the beginning of psychotherapy Carlotta was experiencing intense fear of gaining weight or becoming fat, even though she was seriously underweight. The girl was checking the number of calories consumed by restricting or exercising with the sole purpose of losing weight, but at the expense of friendships, homework, and other responsibilities. Carlotta never referred to having forced vomiting. She met a psychiatrist some time ago but this meeting did not help her, later she began psychotherapy on her own initiative.

During therapy the girl started to specify that her conflict with food was characterized by totally different mood, depending on the day: “Sometime I’m positive and in balance, I can eat pasta or pizza without any problem, but after that, suddenly, it comes out one other me, who make me feel guilty.” Carlotta calls her negative voice by the name of “Rebecca” describing her as “the other part of me – who doesn’t want me to change.” Rebecca, her biggest enemy, makes feel her guilty every time she eat something.

Carlotta chose the name “Rebecca” because it was the name her mother might have given her instead of Carlotta. “Rebecca” forces Carlotta to go running every time she has eaten something, she prevents Carlotta from going out for dinner, she prohibits Carlotta from sitting down to eat and relaxing, without worrying about calories. Rebecca is the personification of the problem. The interaction between the two is perceived as a fight.

### Current study

Overcoming a cognitive approach to motivation for change, in the present study we focused on the “psycho-discursive practices” (Wetherell, 2008) revealing the way in which people frame their will to change. We aim to describe the progress of treatment which focus on the power of metaposition in order to support the dialogical self’s need to change. The metaposition allows the individual to take a “metaperspective” (Hermans, 2006), permitting him or her to evaluate and organize other positions, see the way they are linked, and maintain a vision of the whole, thereby fostering change.

## Case Study

### Texts analyses

The treatment has been developed according to the dialogic self theory by one of the author as a therapist. The transcripts of six therapeutic sessions, lasting 1 h each, have been analyzed by investigating ways in which various discursive changes modify the framing of the will to overcome eating disorder, from the first session (first, second, and third colloquia) to the last (fifth and sixth). We traced the trend toward discursive change during the therapeutic process according to the four dimensions suggested by Hermans et al. (1993).

1. Interactional dominance, consisting of symmetrical or asymmetrical patterns in initiative-response structures. The dominant party “is the one who makes the most initiatory moves. The subordinate party allows, or must allow, his or her contributions to be directed, controlled, or inhibited by the interlocutor’s moves” (Hermans et al., 1993, p. 75).
2. Topic dominance, “one party predominantly introduces and maintains topics and perspectives on topics. By determining the topic of a conversation, an interlocutor may achieve a high degree of dominance that may be visible not only in terms of the content of the talk, but also in terms of the direction that the conversation takes as a whole” (Hermans et al., 1993, p. 75).
3. Amount of talk: which characteristics (number of words, use of open or closed questions) consent to investigate the dominating and the subordinating party (Hermans et al., 1993, p. 76).
4. Strategic movements: any kind of linguistic device which influences the direction and results of discourse; i.e., the use of persuasive, metaphorical language, grammar, or verbal formulas.

We chose a specific pattern of Discourse Analysis with the intention of identifying any linguistic variations which might signal transition from dysfunctional self-narratives to more organized ones. Being inspired by Potter and Wetherell’s (1987) model, our analysis aimed to single out any discursive devices that might reveal the presence of distinct voices amidst the speech of therapy clients. We chose this type of analysis as the most suitable to consider the linguistic aspects defined as the consistent goal of research, our inquiry focused in fact on the structure of the tenses at the syntactical level (Van Dijk, 1998), with particular attention to categories as the pronouns, verbal tenses and forms, adverbs of time, the presence or absence of subordinate clauses, if-clauses, etc. We followed two different pathways. At the utterance level, we collated the multifarious wordings between or among the various speaking positions (or “voices”) of the self, and at the temporal level we contrasted texts produced at the beginning (first, second, and third conversation) and at the end of the therapy (fifth and sixth conversation).

In addition, a modern content analysis was carried out on the texts of the first and the sixth session of therapy using software *Système pour l’Analyse des Données* (SPAD) and a quali-quantitative approach (Lebart and Salem, 1988). These analyses had two aims: first, to ascertain whether the purely hermeneutic “pen and paper” analysis used in the previous phase would be borne out by a more sophisticated computer analysis; second, to identify from the texts a specific lexicon in relation to the voice of the client occupying the scene in the dialog (Carlotta-Rebecca-Metaposition) and in relation to the time of the interview (beginning, end of therapy).

The analysis was carried out in order to identify vocabulary which was more characteristic of one voice of the individual with respect to another, and to trace out a change in the client’s style of response, from one time to the next (Murakami, 2010; Faccio et al., 2011b, 2012b). The Vospec (specific vocabularies) procedure from the software Spad was applied to the texts, which were pre-treated so as to define the precise usage of particular terms. This involved:

1. keeping homographs separate and grouping synonyms together;
2. eliminating words considered irrelevant to the analysis following three main criteria: (a) words with no significance in relation to the research objectives were eliminated (e.g., conjunctions and prepositions such as but, and, with etc.); (b) low frequency words were eliminated (one occurrence or less); (c) words unrelated to the specific research questions were eliminated;
3. creating equivalences among different verbs having the same tense. In this way, we created categories distinguishing positive and negative modal verbs, verbs in the imperative, conditional, past, and present indicative;
4. creating equivalences by uniting nouns used to describe emotional states, which were classified either as “positive emotions” or “negative emotions.”

Through the Vospec procedure, specificity measures were obtained indicating to what extent certain words were characteristic of one group compared to the other. The analysis generated frequency tables, such as that reported in the Results section below, showing the characteristic words of Carlotta’s voice compared to Rebecca’s voice and the Metaposition, as well as the vocabulary typical of the first section compared to the last section of the therapy (the terms are listed in order of significance; the column “global frequency” indicates how often the words appear in the entire documents; the column “internal frequency” indicates how often the words appear in a specific group). The value test measures the deviation between the percentage of a graphical form in a class and its total percentage. The significance level is fixed at *p* < 0.05.

## Personal Narratives

The presentation of the results of the discursive analysis is organized in four sections. The first two sections report the analyses of the four dimensions suggested by Hermans et al. (1993) at the beginning and at the end of the treatment, respectively. In the third section a comparison between these two phases is conducted by highlighting the role of assuming a metaposition in order to grasp the therapeutic function. Finally, in the fourth section the discursive construction of change during therapy is presented.

### Discursive analysis of the sessions at the beginning of therapy

#### Interactional dominance in the first clinical session

Rebecca’s voice is definitively predominant. In terms of the initiative-response structure, Rebecca’s voice prevails over Carlotta’s, who says as example: *“I know Rebecca and I know what happens afterward. To avoid suffering afterward, I avoid beforehand.”*

Rebecca sometimes predicts Carlotta’s actions and prevents them. Carlotta is directed, controlled, or inhibited by Rebecca’s moves. Nevertheless, the other party is still able to have her say, limited as that may be, and at times manages to fight against the dominant voice: *“There are days when I’m really positive, really happy and so I say ‘I’m going to beat Rebecca’, other days I’m a bit sadder and a bit more tired and worried and so she wins.”*

By way of linguistic analysis, we can affirm that the speaker here does not identify with Rebecca: the voice uses the third person singular and addresses Carlotta with “you” (the second person singular). In contrast, Carlotta’s voice speaks in the first person singular and always in the present-tense.

#### Topic dominance in the first clinical session

It is Rebecca who predominantly introduces and maintains topics and perspectives. The dominance is visible not only in terms of talk content, but also in terms of the direction that the action then takes: *“Look! Now you’ve eaten the pizza and now you have to stay behind.”*

Rebecca only has one topic, that of anticipating or making up for a dieting “slip-up,” but it is more important than anything Carlotta thinks. Carlotta is especially restricted because the dominating party does not require an answer, but only obedience: *“You have to make up for what you did that day”; “Do it! All you have to do is go jogging*.”

#### Amount of talking and strategic moves in the first clinical session

Carlotta says: *“nine out of 10 times Rebecca jumps out.”* Such restricted responses may also be the result of the interlocutor’s prestige or style of questioning (suggestive of answers or Socratic method). It is not necessary to talk a great deal: when someone says few things, but strategically important, the direction and the resulting insights may be heavily influenced.

Rebecca says few strategically important things, she uses logical, rational strategies, based on convincing demonstrations of cause-effect: *“There, now you’ve eaten pizza, so tomorrow you’ll have to cut back because without a doubt you’ve put on a lot of weight.”* Even the temporal adverbs that characterize her speech (*“now get up,” “tomorrow go jogging,” “yesterday you ate”*) signify that only one definition of time is possible: the present is planned in relation to the past and future. Conditional clauses and imperative forms (*“you can eat if you go jogging tomorrow”*) contribute to giving the dialog a sense of necessity.

As confirmed by the SPAD analyses, as Table 1 shows, Rebecca’s voice uses mainly verbs in the imperative mode, or verbs indicating necessity, command (you must go jogging, you must stay behind, you must do, stay on a diet, go running…); they form propositions which bind Carlotta to experiences of obedience and restriction. In the vocabulary attributed to Rebecca, there also are abundant instances of disapproval (that’s not good, don’t go running, your belly has gotten fatter…) and a recurrence of negative moods (disappointment, disturbance, fear, guilt feelings, sense of duty, anger…).

**Table 1:** **Specific vocabulary from fragments of conversation attributed to the voice of Rebecca**.

Characteristic words or segments	Internal frequency	Global frequency	Test-value	*p*
Imperatives	17			
Instances of disapproval	6	9	2.222	0.013
Negative emotions	12	29	1.599	0.055

Carlotta does not employ such refined strategies, her speech is characterized by modal verbs that, rather than strengthening each other, weaken each other: *“I tried to have a go at*…,*”* *“I manage a bit more to*….” Most importantly, the SPAD analyses show that at the beginning of therapy Carlotta mainly chooses negative forms when using certain modal verbs (*I cannot fight, I cannot imagine, I cannot control*…).

Here is a grammar of the “indefinite”: *“before,” “after,” “for a few months,” “a bit more,” “I used to eat anything without problems anytime I wanted to.”*

In these early exchanges between Rebecca and Carlotta we perceive no true dialog, but rather, Rebecca’s imposition over Carlotta. Rebecca is at the top of the hierarchy: she polarizes and dominates the other voices.

### Discursive analysis of the sessions at the end of therapy

#### Interactional dominance at the end of therapy

During the final sessions of the therapy Rebecca makes herself heard less and Carlotta manages to behave differently with regard to food.

The voice of the Metaposition makes the most initial moves, and then, a little at a time, becomes the mediator of the dialog between the parts and the privileged interlocutor for Carlotta.

Sometimes the Metaposition prefers coordinate to subordinate clauses; she doesn’t use the imperative form. We no longer have a “monolog” by Rebecca, but a symmetrical dialog; the voice of the Metaposition occupies a higher hierarchical position from which to organize the exchange, favoring reciprocal interaction.

#### Topic dominance at the end of therapy

Rebecca tries to introduce and maintain topics and perspectives; she has not disappeared, but now the equilibrium has changed: dialog with the Metaposition sustains her.

#### Amount of talking at the end of therapy

Rebecca now talks less – *“I haven’t heard from her for 7 days”* – whereas the Metaposition makes herself heard frequently.

#### Strategic movements at the end of therapy

Through the use of a particular style of questioning, the direction and the resulting insights may be heavily influenced. The Metaposition often requires only a “yes” or “no” answer, or brief replies (Hermans et al., 1993). In general, in the latter part, a question-answer type of dialogical structure prevails in the mediating voice of the Metaposition.

Finally, if we compare Tables 2 and 3, we see that at the start of therapy the use of negative modals prevailed (I cannot fight, I can’t imagine, I can’t control), constructing a position identifying the individual in such a way that she experience herself as incompetent; a person swayed by continual failures as she attempts to keep her resolutions. The abundant use of conditionals (I would have, I could, I would like, I would do…) clearly reflects a tendency to establish wishes and intentions concerning one’s behavior (I could eat less, I’d like to do this…), only to find oneself unable to respect them in practice. Such akratic experiences of failure become a source of guilt for Carlotta, and they end up feeding the severity and the escalation of demands developed by Rebecca. As noted earlier, in fact, the negative voice makes abundant use of disapproval (that’s not good, don’t go jogging, your belly has gotten fatter…) and of imperatives (you must go for a run, you must stay behind, you must do, stay on a diet, go running…). The most harmful effect of such rhetorical strategies is to overshadow the other voices and to prevent any dialogical exchange between the positions.

**Table 2:** **Specific vocabulary from fragments of conversation at start of therapy**.

Characteristic words or segments	Internal frequency	Global frequency	Test-value	*p*
Negative modals	8	11	2.078	0.019
Instances of disapproval	5	9	0.773	0.220
Condizionale	9	19	0.650	0.258
Imperatives	17	39	0.620	0.268

**Table 3:** **Specific vocabulary from fragments of conversation at end of therapy**.

Characteristic words or segments	Internal frequency	Global frequency	Test-value	*p*
Present	61	79	3.024	0.001
Emotions: positive	18	26	0.543	0.294
Modals: positive	17	26	0.121	0.452

At the end of treatment, instead, we see that in the client’s speech the use of present-tense verbs become prevalent (I do, I think, I want, I see, I feel…) along with positive modals and past-tense verbs (I recovered, I decided, I stopped doing, I can ask myself, I can say, I can…). These linguistic constructions denote the renewal of a sense of “authorship”; and of the possibility to re-narrate one’s experience in virtue of a retrieved decision-making capacity and a more solidly formed sense of control. Finally, on the level of personal experience, whereas at the start of treatment we perceived a prevalence of stress-causing emotions, now there is more room for positive emotions (strength, happiness, tranquil, serene, secure, feel like laughing…). These are sometimes defined deliberately in relation to the absence of a negative mood, which was present at first but has been overcome during the course of therapy (less controlled, less rational, without feeling guilty, without having to…).

### Comparison between the beginning and end of therapy

During the final treatment sessions, the mode of interaction between the voices changes thanks to a third “voice” that was already present, albeit very weakly so: the metaposition. It uses reflexive verbs (*“I surprise myself,” “I control myself”*) and it has, grammatically speaking, a “reflexive function.” The auxiliary verbs “to have to” and “to want to” are contrasted by an increasingly intense use of verbs expressing the person’s condition (*“I’m well”*) and sensations (*“I feel okay,” “you don’t like”*); the prevalent tense is the past, which the speaker uses in reflecting about the differences now emerging in her current situation (*“I understood that*…,*” “I allowed myself,” “once I used to say*…*”*).

The SPAD analyses (Table 4) also show that the voice of “I the therapist” uses mostly present-tense verbs (*I do, I think, I want, I see, I feel*…) and positive modals or past-tense verbs (*I recuperated, I decided, I stopped doing, I can ask myself, I can say, I can*…) which indicate the affirmation of an active role in her experience, and a consequent increase in the perceived sense of self-sufficiency.

**Table 4:** **Specific vocabulary from fragments of conversation attributed to the voice of “I-therapist**.”

Characteristic words or segments	Internal frequency	Global frequency	Test-value	*p*
Present	55	79	4.458	0.000
Positive modals	16	26	1.357	0.087

The verbal formula “be + gerund” marks the stages of a journey toward change, which is in progress: *“I’m making a journey,” “I’m realizing that I’m able to follow my feelings,” “Carlotta is coming back.”* This voice seems strong enough to oppose the dominant position of Rebecca, and to effectively reorganize the self (Hermans and Dimaggio, 2007).

## Discussion

A little at a time, the metaposition starts to use the first person singular (*“I tell myself*…*”*), thus assuming an increasingly important role in the hierarchical organization of Carlotta’s dialogical self. Nevertheless, it utilizes its dominance in a functional way, favoring dialog, and mediating between the other positions. There is a more symmetrical relationship between Rebecca and Carlotta: both have the right to speak and be listened to, but the last word goes to the voice that speaks from the metaperspective. For example: the psychotherapist defines the qualities of the day using neither Carlotta’s criteria (for her the day is “good” if she has eaten), nor those of Rebecca, for whom the day may be “empty, full of nothing”; she defines it merely as “particular.” The mediating voice also manages a new flexibility when shifting from one position to another. “I won’t let Rebecca do whatever she wants to me anymore. (…) Lately I’ve been transforming the negative things that Rebecca wants to tell me and playing them to my favor. The relationship with Rebecca has really changed.”

In more general terms, an analysis of the four different dimensions involved in dialog with the dominant element allows us to light up the passage from a condition of dysfunctional self-narratives to more organized ones (in Hermans’ terms). In the earlier condition the voices speak in monolog, in a strong, rigid hierarchy of self-positions. In this condition the potential for dialog is limited by a dominant voice. To facilitate a different organization of repertoires for I-positions would be crucial to the emergence of contra-positions or meta-positions.

To summarize: self-narratives emerging in the earlier session are disorganized and monological in form (Hermans, 2006). They show: a strong, rigid hierarchy of I-positions, in which Rebecca’s position is dominant; a limited capacity for dialog between voices; rigid interpretation, and construction of experiences. The other positions (Carlotta, the metaperspective and others) are constantly pushed into the background; they do not participate in the dialogical process.

The self-narratives emerging in the latter session are better organized (Hermans, 2006). They show the emergence of a contra-position, i.e., the metaperspective. It is strong enough to contrast the Rebecca position. Even in this more organized system, one position is dominant over another (the hierarchy still applies); but the dominance becomes relative: voices take up a dialog with other voices, negotiating meaning; they alternate in adaptive ways, under supervision by the metaposition; dominance becomes intrinsic in organizing the repertoire of positions.

## Conclusion

Our study is based on the premise that by itself, the motivational construct may be insufficient in explaining the dynamics that involve the client who is deciding whether or not to commit himself to a process for change. In imagining the individual as inhabited by different voices of his consciousness, instead, we can see that in expressing the problem, various parts of the self may exist in disaccord with one another. Some of them may keep the client placed within a regime of non-change. Others, though undergoing domination by rigid, judgmental parts of the self, may, instead, become the promoters of a “motivated” request for change.

In distinguishing the voices at play in maintaining the problem, and at play, as well, when the client requests change, we have attempted to shed light on several linguistic patterns which may reveal the passage from one position to another. The results of our analysis also show that such patterns may be employed both in characterizing the “psychological profile” of various self-positionings, and to identify the particular interchange made possible between or among them.

In light of the theory of the dialogical self, we can, then, understand the client’s evolution during therapy as an improvement in dialog between or among the various parts. Moreover, based on results emerging from this study, we would also suggest that the improvement can be widely demonstrated (and clearly documented) by the therapist simply by listening carefully to the particular lexicon used by the client in describing his or her experience.

In this connection, the analysis of discourse carried out on a purely qualitative level on the text of interviews during treatment proved sufficient for identifying recurrent linguistic patterns, even at the treatment site. Instead, as regards integral analyses carried out using SPAD, although the results emerging here may appear uncertain due to the briefness of the texts analyzed, the same procedures might constitute a satisfactory means for exploring the lexicon characterizing a wide range of clinical cases. It would be interesting, indeed, to trace out a “language of change,” by determining, e.g., which vocabularies or grammatical forms are most easily associated with maintaining rigid positions, or with rhetorical devices that discourage a dialogical exchange between and among parts of the self.

Clinical psychology has to address bulimia developing specific protocols and models (Manzoni et al., 2008; Castelnuovo, 2010a,b; Pietrabissa et al., 2012) above all in new settings (Molinari et al., 2012) and technology-based scenarios (Castelnuovo et al., 2003a,b, 2010, 2011a,b,c; Castelnuovo and Simpson, 2011; Manzoni et al., 2011), improving not only evidence-based prescriptions (Castelnuovo et al., 2004, 2005, 2008; Castelnuovo, 2010b), but also the “language of change” that is an important clinical resource very different from the typical language used in scientific contexts (Castelnuovo et al., 2008).

## Conflict of Interest Statement

The authors declare that the research was conducted in the absence of any commercial or financial relationships that could be construed as a potential conflict of interest.
